# Study on the key ecological factors for the reproduction of *Gymnocypris eckloni* in the upper reaches of the Yellow River

**DOI:** 10.1002/ece3.70202

**Published:** 2024-08-22

**Authors:** Lihao Guo, Guodong Li, Qiaoling Zhang, Guoyong Zhang, Weiying Wang, Zijun Liu, Shanshan Li

**Affiliations:** ^1^ State Key Laboratory of Eco‐Hydraulics in Northwest Arid Region of China, School of Water Resources and Hydropower Xi'an University of Technology Xi'an China; ^2^ China Renewable Energy Engineering Institute Beijing China

**Keywords:** aquatic ecology, dam‐induced spawning ground restoration, habitat protection, habitat simulation, reproduction ecology

## Abstract

The development of hydroelectric projects has adversely affected the reproductive activities of downstream fish species. To facilitate the natural reproduction of fish and restore spawning grounds post‐dam construction, it is imperative to explore the ecological factors crucial for their reproduction. Currently, various research methods with different advantages and limitations are employed for this purpose. Using identified spawning locations and periods as clues, we quantitatively investigate the flow velocity, water depth, water temperature, and riverbed substrate required for spawning. The results are validated using habitat simulation methods, aiming to establish a more scientific approach to explore ecological factors affecting fish reproduction. This study provides a more scientific, systematic, and detailed report on the ecological factors required for the spawning of *Gymnocypris eckloni*: flow velocity ranging from 0.19 to 0.97 m/s, water depth from 0.28 to 1.12 m, water temperature between 11.4 and 15.2°C, and predominantly gravel substrate. The reliability of the results was verified in another spawning ground, with good verification results. This research provides crucial data for the bio‐mimetic reproductive technology of *Gymnocypris eckloni* and the restoration of spawning grounds for natural fish reproduction post‐dam construction. It addresses the lack of suitable ecological factor data for protective fish species in the upper reaches of the Yellow River. The method exhibits strong scientific, accurate, and implementable characteristics.

## Graphical abstract

This study quantitatively determined the demand for reproductive environmental factors of *Gymnocypris eckloni*, which is of great significance for its population and habitat protection.

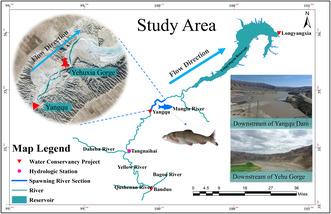

## 1 INTRODUCTION

Dams play a critical role in the development of rivers worldwide, providing essential support for flood control, power generation, water supply, and navigation (Yüksel, 2010). However, the construction of such infrastructure alters the natural morphology of river channels. Primarily, dam construction modifies natural runoff processes, leading to a flattening of the runoff regime (Poff & Schmidt, 2016). Additionally, the hydropower peak regulation causes significant intraday flow fluctuations, resulting in frequent water level changes downstream of the dam, greatly increasing the likelihood of fish eggs drying out and becoming nonviable (Bipa et al., 2024). The sediment trapping effect of dams, combined with new water release mechanisms, reshapes downstream river channels (Best, 2019), and hydrodynamic conditions (such as flow velocity; Fellman et al., 2019 and water depth; King et al., 2016) also change with the altered topography. The establishment of reservoirs reduces flow velocity within the reservoir, causing sedimentation of pollutants and affecting downstream water quality (Chen et al., 2020; Maavara et al., 2020). The considerable depth of reservoirs can lead to thermal stratification, resulting in different water temperatures compared to natural inflows (Tang et al., 2006). These factors are intimately connected to various aspects of fish reproduction, with each alteration potentially impacting fish spawning (Cooper et al., 2017; Stone, 2016).

The *Gymnocypris eckloni* (*G. eckloni*) is an important aquatic wild species in the upper reaches of the Yellow River, listed as a provincially protected species. The study area is a critical spawning ground for this species, and the construction of hydropower stations will impact its population (Han, 2016). Understanding the *G. eckloni* sufficiently before the emergence of factors threatening its population is essential for future conservation efforts. As early as 1988, the *G. eckloni* caught the attention of scholars. Wang (1988) dissected it and compared its olfactory organs with those of the *Gymnocypris przewalskii* from Qinghai Lake. In 2002, Li et al. (2002) conducted mitochondrial restriction enzyme analysis on *G. eckloni* in Lake Tuosuo. In 2009, Qi (2009) analyzed the sequence variation and genetic diversity of the Cyt b gene in *G. eckloni*. The existing research primarily focused on molecular biology and genetics, elucidating the intrinsic differences within the population. This work filled a significant gap in the study of the *G. eckloni* and served as a good starting point. However, it did not address the reproductive aspects of the *G. eckloni*, and therefore, these studies cannot be directly applied to habitat conservation efforts. Until 2016, Yan (2016) conducted a systematic study on the age, growth, and population genetic characteristics of *G. eckloni* in the upper Yellow River, being the first scholar to focus on the reproductive characteristics of the species. This study described the species as producing adhesive demersal eggs, inhabiting water temperatures of 9–14°C, with spawning occurring from April to July, peaking in May, and qualitatively described the spawning ground depth as around 1 meter. It mentioned the relevant spawning times, water temperatures, and water depths, marking the first description of the spawning characteristics of *G. eckloni* in the upper Yellow River. However, the data were not comprehensive and lacked sufficient evidence to support the findings, failing to meet the data needs for habitat conservation efforts.

In summary, it can be observed that there are few literature reports on the *G. eckloni*, with most focusing on molecular biology. Reports on the environmental requirements for spawning are even rarer, often based merely on experiential descriptions without scientific evidence. Therefore, to protect the critical stages of the *G. eckloni* population's continuation, this paper systematically explores the spawning environmental factors of the *G. eckloni* using comprehensive scientific research methods. This study primarily determines the detailed spawning times and more precise spawning patch locations through investigations. It then analyzes the flow variations during the spawning period. Through numerical simulations, it identifies the flow velocity and water depth at the spawning patch locations. Long‐term water temperature monitoring facilities are installed to obtain the spawning period's water temperature. Additionally, aerial photography and field surveys are used to determine the substrate conditions required for spawning. The results are applied to another spawning site to validate their accuracy, offering a more scientifically grounded method for acquiring environmental factors related to fish spawning. Simultaneously, the research advances the understanding of natural reproduction in *G. eckloni*, holding significant implications for ecological management, spawning ground restoration, and scientifically guided stock enhancement efforts in the post‐dam construction period.

## 2 METHODS AND MATERIALS

### 2.1 Study area

The Yellow River, often referred to as the “Mother River” in China, is the country's second‐longest river, spanning a total length of 5464 km. It stands as one of the regions with the richest water resources in China, making it a vital contributor to the nation's hydroelectric power. Additionally, it is recognized as an essential “genetic bank” for various species (Jiang et al., 2016), hosting approximately 143 fish species (Shen et al., 2014). The Yellow River plays a pivotal role in sustaining global biodiversity and providing genetic resources for the development of fisheries. Flowing through nine provinces and divided into upper, middle, and lower reaches, the river's upper reaches, specifically from the source to the Longyang Gorge in Qinghai, are designated as the river source segment. This area is one of the few regions in the upper reaches of the Yellow River where the ecological preservation of several fish species is effectively maintained.

**FIGURE 1:**
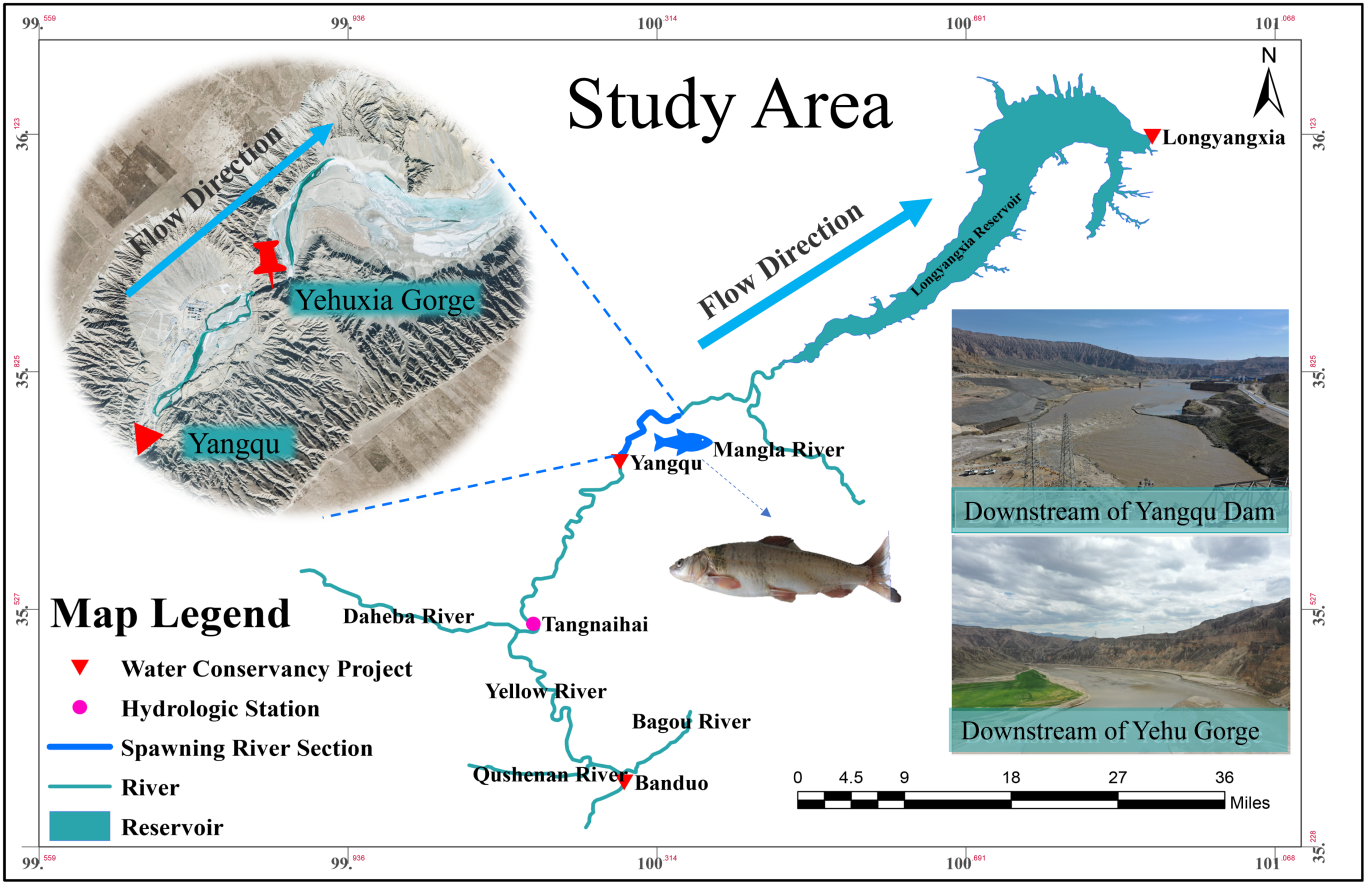
Study area map.

The Yangqu Hydropower Station (35°42′45.01″ N, 100°16′3.45″ E) is located at the border of Xinghai County and Guinan County in Hainan Prefecture, Qinghai Province, China. Positioned upstream of the Banduo Hydropower Station and downstream of the Longyangxia Hydropower Station, it represents a typical wide valley river segment within the ecosystem. This area is characterized by a rich distribution of unique fish resources in the upper reaches of the Yellow River (Shen et al., 2014). It serves as a crucial spawning ground for fish, with historical survey data indicating dominant species such as *Gymnocypris eckloni*, *Acanthogobio guentheri*, and *Triplophysa siluroides* (Li et al., 2020). The selected spawning section for this study spans a total length of 5.9 kilometers (indicated by the blue line segment in Figure 1) and primarily includes two fish spawning areas: the downstream section of the Yangqu Dam and the downstream section of the YeHu Gorge. The Yangqu Dam downstream section extends for 2.4 km, while the YeHu Gorge downstream section covers a length of 2.5 km. The YeHu Gorge itself, situated between the two, has a length of 1 km. An overview of the study area is depicted in Figure 1.

### 2.2 Determination of fish species for study

**FIGURE 2:**
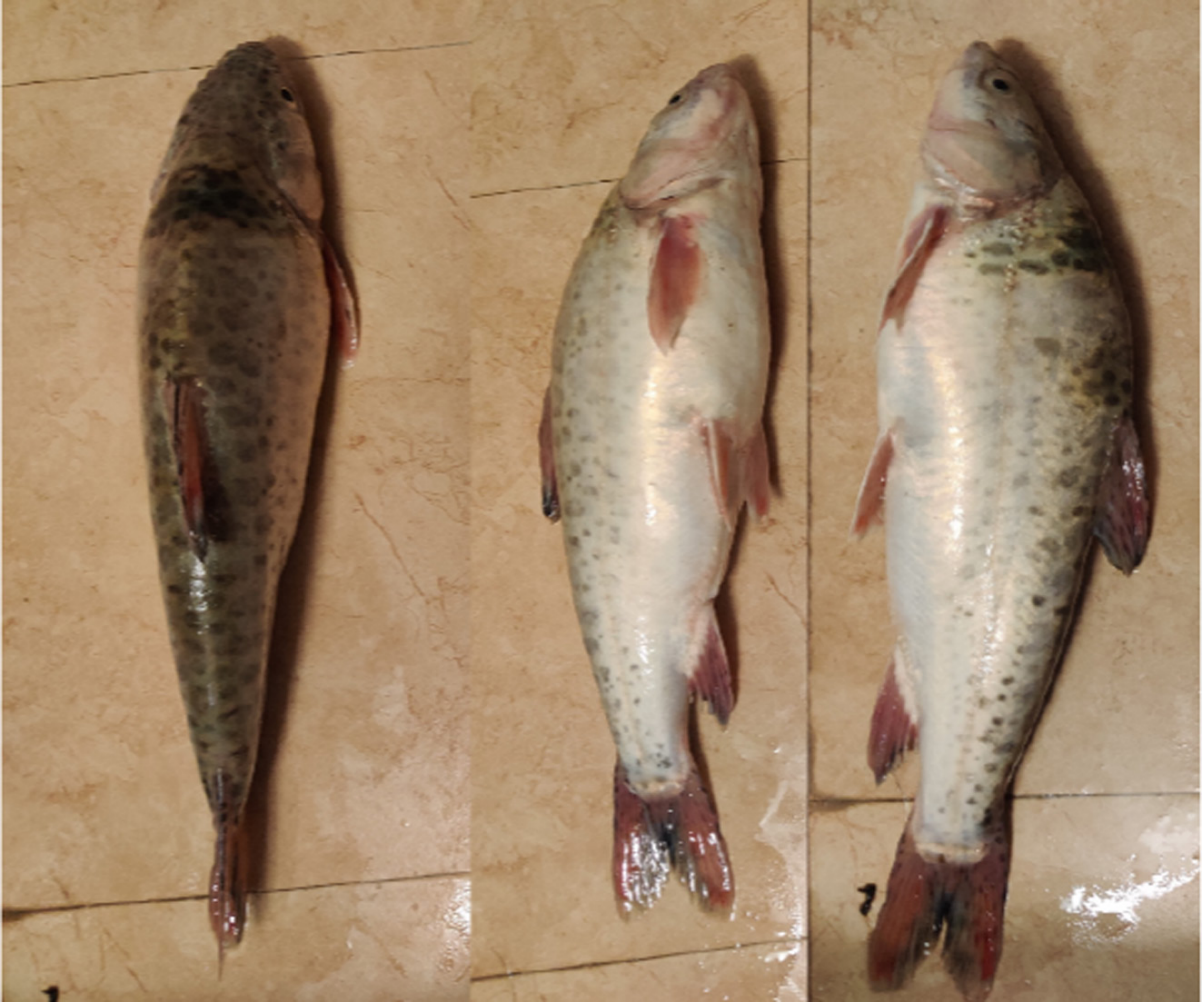
Photograph of *Gymnocypris eckloni*.

The upper reaches of the Yellow River are predominantly inhabited by seven indigenous fish species, including the *Gymnocypris eckloni*, *Gymnodiptychus pachycheilus*, *Platypharodon extremus*, *Acanthogobio guentheri*, and *Triplophysa siluroides*, which are rare or unique species. Most of these fish species exhibit an adhesive‐spawning type of reproduction (Shen et al., 2014). Among them, *G. eckloni* (Figure 2), belonging to the Cyprinidae family in the Cypriniformes order, Schizothoracinae subfamily, is characterized by its elongated and flattened body with a pointed head. It is mainly distributed in the upper reaches and tributaries of the Yellow River, lakes such as Zhaling Lake and Eling Lake. *G. eckloni* is a unique fish species in the upper reaches of the Yellow River (Han, 2016) and serves as the dominant species in the study area. Many researchers choose it as an indicator species for the aquatic ecosystem in this river segment (Jiang et al., 2005). According to “Medicinal Fauna of China,” *G. eckloni* has significant medicinal value, with its gallbladder, bones, and meat being used in traditional medicine (Li et al., 1983). It holds substantial research value and significance.

### 2.3 Investigation of spawning ground environmental factors

**FIGURE 3:**
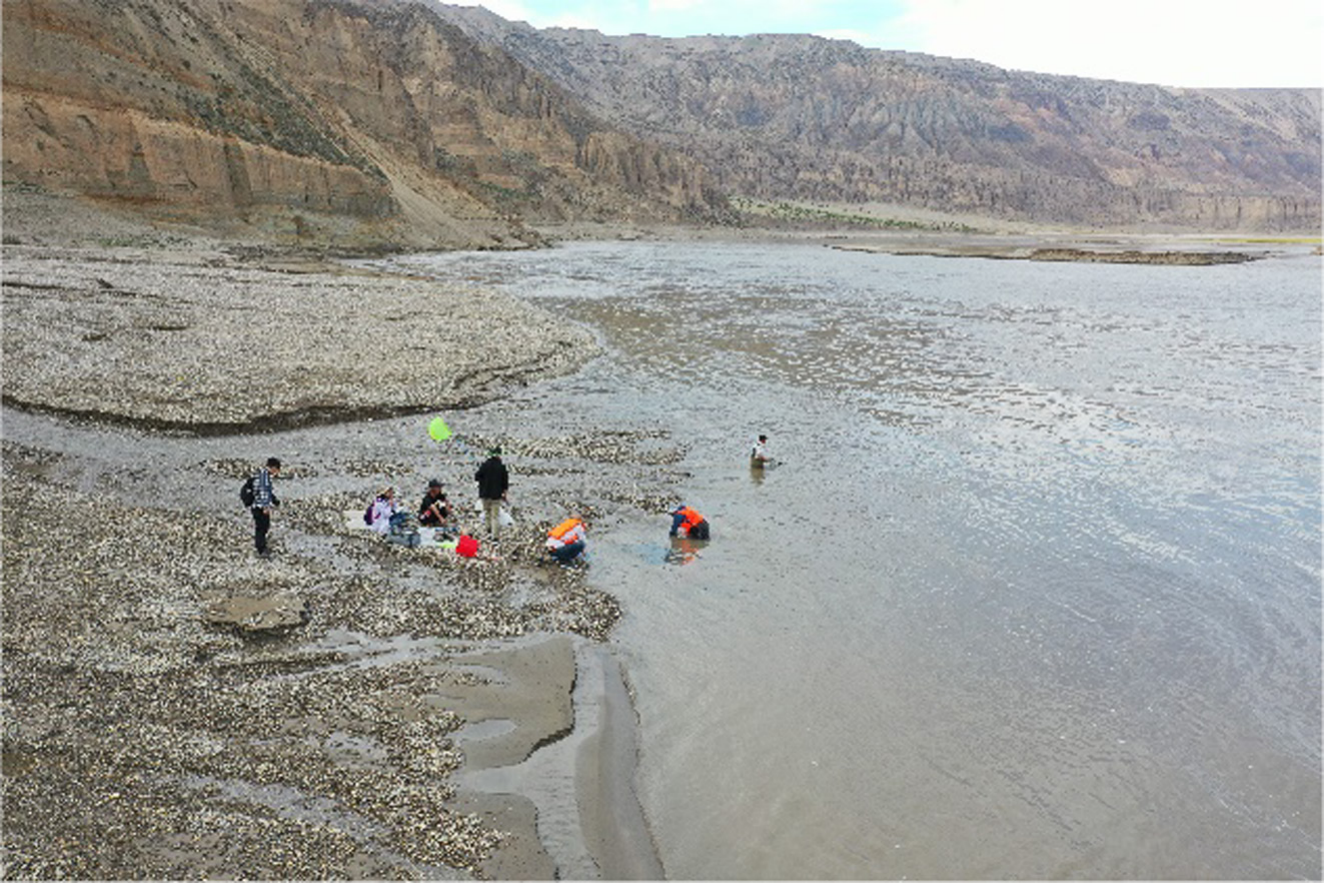
Field investigation site.

The unique terrain and hydrological features within the study area provide crucial guarantees for the continuity of fish populations from one generation to the next. The distinct hydrodynamics, water temperature, and substrate conditions in this region hold special significance for fish reproduction. This study conducts onsite investigations (Figure 3) to identify the primary spawning locations of *G. eckloni* and utilizes various methods to explore the unique hydrodynamics, water temperature, and substrate conditions at these identified locations. This aims to clarify the quantitative values of the factors influencing fish spawning.

The specific methodologies are outlined below.

#### 2.3.1 Hydrodynamic factors

Hydrodynamic factors are crucial conditions ensuring the smooth spawning and embryonic development of fish (Lechner et al., 2014). Traditional observation methods often involve measuring random points within spawning grounds during surveys or calculating cross‐sectional averages for the entire spawning area. Manual measurements, being somewhat random, may not fully capture the overall hydrodynamic characteristics of fish spawning locations. Additionally, defining fish hydrodynamic requirements based on cross‐sectional averages can lead to overestimations. To address these issues, this study employs a two‐dimensional hydrodynamic model to simulate the hydrodynamic conditions within the spawning grounds. It calculates the day‐to‐day hydrodynamic conditions at the spawning locations of *G. eckloni* during the reproductive period. The model computes and averages the flow velocity and water depth data for the region, providing a more accurate reflection of the overall hydrodynamic features within the spawning area. The terrain data are obtained through onsite measurements, and the model's input uses the daily flow rate measured at the Tangnaihai hydrological station, with the outlet boundary set as the water level.

The numerical simulation primarily employs the MIKE21 numerical model based on the shallow water equations (Warren & Bach, 1992). Two‐dimensional hydrodynamic models for the downstream section of the Yangqu Dam and the YeHu Gorge are established using a set of governing equations, including the continuity equation and momentum equations in the x and y directions:

(1) $$\frac{\partial \zeta }{\partial t}+\frac{\partial p}{\partial x}+\frac{\partial q}{\partial y}=\frac{\partial h}{\partial t}$$

(2) $$\begin{array}{l} \frac{\partial p}{\partial t}+\frac{\partial }{\partial x}\left(\frac{p^{2}}{H}\right)+\frac{\partial }{\partial y}\left(\frac{\mathrm{pq}}{H}\right)+\mathrm{gH}\frac{\partial \zeta }{\partial x}+\frac{\mathrm{gp}\sqrt{p^{2}+q^{2}}}{C^{2}H^{2}}-\\ \frac{1}{\rho }\left[\frac{\partial }{\partial x}\left(H\tau_{\mathrm{xx}}\right)+\frac{\partial }{\partial y}\left(H\tau_{\mathrm{xy}}\right)\right]-\mathrm{fq}-f_{w}\left|W\right|W_{x}=0 \end{array}$$

(3) $$\begin{array}{l} \frac{\partial p}{\partial t}+\frac{\partial }{\partial y}\left(\frac{q^{2}}{H}\right)+\frac{\partial }{\partial x}\left(\frac{\mathrm{pq}}{H}\right)+\mathrm{gH}\frac{\partial \zeta }{\partial y}+\frac{\mathrm{gp}\sqrt{p^{2}+q^{2}}}{C^{2}H^{2}}-\\ \frac{1}{\rho }\left[\frac{\partial }{\partial y}\left(H\tau_{\mathrm{yy}}\right)+\frac{\partial }{\partial x}\left(H\tau_{\mathrm{xy}}\right)\right]-\mathrm{fq}-f_{w}\left|W\right|W_{y}=0 \end{array}$$

In the equations, where *H* represents water depth, *H = h + ζ*, with ζ and h denoting water level and water depth, respectively, measured in meters (m); *p* and *q* are the flow flux in the *x* and *y* directions, measured in cubic meters per second (m^3^/s); *C* is the Chezy coefficient, m^1/2^/s; *g* stands for gravitational acceleration, m/s^2^; ƒ is the Coriolis force coefficient; *ρ* represents the density of water, kg/m^3^; *W*, *W*_*x*_, and *W*_*y*_ denote wind speed and its components in the *x* and *y* directions, m/s; ƒ_*w*_ is the wind resistance coefficient; *τ*_*xx*_, *τ*_*xy*_, and *τ*_*yy*_ represent the components of effective shear forces, *N*.

#### 2.3.2 Water temperature

**FIGURE 4:**
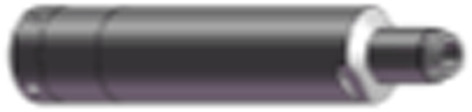
Schematic drawing of high‐precision temperature probe.

Water temperature plays a crucial role in the process of fish spawning, as suitable temperatures stimulate the production and release of eggs and sperm, representing a prerequisite for successful fish reproduction. While water temperatures in river channels exhibit relatively small variations within a certain range, temperatures at different times fluctuate with changes in air temperature (Pu et al., 2006). Traditional methods of water temperature observation involve single‐point measurements, resulting in incomplete results that fail to encompass the entire range of suitable temperatures for fish reproduction. In this study, high‐precision temperature probes (Figure 4) were strategically placed in fish spawning grounds, equipped with network devices for hourly reporting of water temperatures. This approach ensures a more accurate representation of the suitable temperature range for fish reproduction. The temperature probes operate within a temperature range of −45 to 130°C, with an error margin of ±0.1°C and a resolution of 0.01°C.

#### 2.3.3 Substrate

Substrate serves as the primary physical environment for fish spawning and embryonic development, providing the foundational matrix for fish reproduction. Previous research on substrate primarily employed two methods: substrate sampling and sieving, and detection using underwater cameras. The first method is constrained by the size limitations of collection instruments, rendering it ineffective for substrates exceeding the instrument dimensions. The second method is an ideal analytical approach, but it is hindered by the low water transparency of the Yellow River, preventing optical cameras from effectively detecting substrates.

In this study, the spawning substrate for *G. eckloni* was confirmed through two surveys: one during the fish spawning period and another during the low‐flow period. The first survey, conducted during the fish spawning period, faced challenges due to the low visibility of the Yellow River's water, providing limited opportunities to collect substrate samples from the spawning grounds. However, the substrate distribution obtained during this period best reflects the actual requirements for the fish spawning substrate. The second survey, conducted during the low‐flow period, aimed to further obtain detailed information on substrate distribution, such as grain size and the proportion of different substrate types, building on the findings from the first survey. During the low‐flow period, substrate data collection primarily involves a combination of aerial photography and onsite surveys, covering the entire target river segment. Aerial photography is used to construct a two‐dimensional digital model of the entire river channel, providing an overview of the general substrate distribution in the region. For key spawning areas, onsite investigations are conducted, and substrate composition is analyzed using 1 m × 1 m sampling frames.

#### 2.3.4 Results validation

This study primarily focuses on the spawning grounds below the Yangqu Dam as the application scenario for obtaining the environmental factor requirements of *G. eckloni*. The downstream area of the YeHu Gorge serves as the validation scenario, and the validation employs the Habitat Suitability Index (HSI) method within the Habitat Simulation method. This method establishes the suitability index for each indicator through field surveys or laboratory experiments. The HSI combines the results from numerical simulations to obtain the suitability distribution of various indicators within the research river segment. The comprehensive method for the suitability of each indicator in this paper is based on the minimum value method:

(4) $${\mathrm{HSI}}_{i}=\mathrm{Min}\;\left({\mathrm{SI}}_{d}\cdot {\mathrm{SI}}_{v}\cdot {\mathrm{SI}}_{s}\cdot {\mathrm{SI}}_{T}\right)$$

Here, ${\mathrm{SI}}_{d}$, ${\mathrm{SI}}_{v}$, ${\mathrm{SI}}_{s}$, and ${\mathrm{SI}}_{T}$, respectively, represent the suitability indices for water depth, flow velocity, substrate, and water temperature.

### 2.4 Date collection and processing

This study utilized four main types of data, namely terrain, hydrological, water temperature, and aquatic data. The hydrological and terrain data were employed for hydrodynamic simulation, while water temperature and aquatic data were used to analyze environmental factor requirements.

**FIGURE 5:**
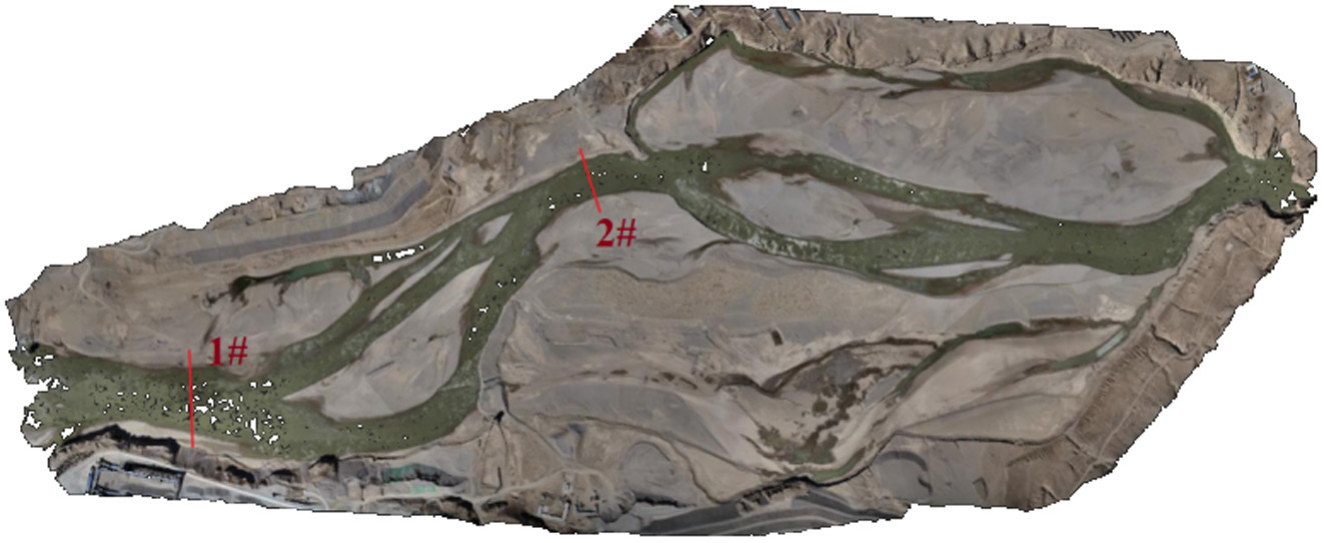
Model validation for hydrological cross‐section positions.

Before conducting hydrodynamic simulations, the model required parameter calibration and validation to ensure its accuracy. Hydrodynamic simulation and validation in this study utilized terrain data collected by our team in March 2023. The model validation employed water level section data surveyed during the same period, and the locations of these sections are depicted in Figure 5. The boundary hydrological data used for simulation calculations were obtained from the Tangnaihai hydrological station. Water temperature data were collected using automatic monitoring devices installed by our team during the same period. The spawning period and spawning locations were determined based on the investigations conducted by our team in the respective year.

It is important to emphasize that, for the hydrodynamic simulation of the spawning grounds upstream and downstream of the YeHu Gorge, the measured flow rates from the Tangnaihai hydrological station were used as the inlet boundary conditions. The outlet boundary conditions were determined by calculating and extracting the water level at the outlet from the Yangqu‐Longyangxia reservoir section.

## 3 RESULTS

### 3.1 The spawning locations

**FIGURE 6:**
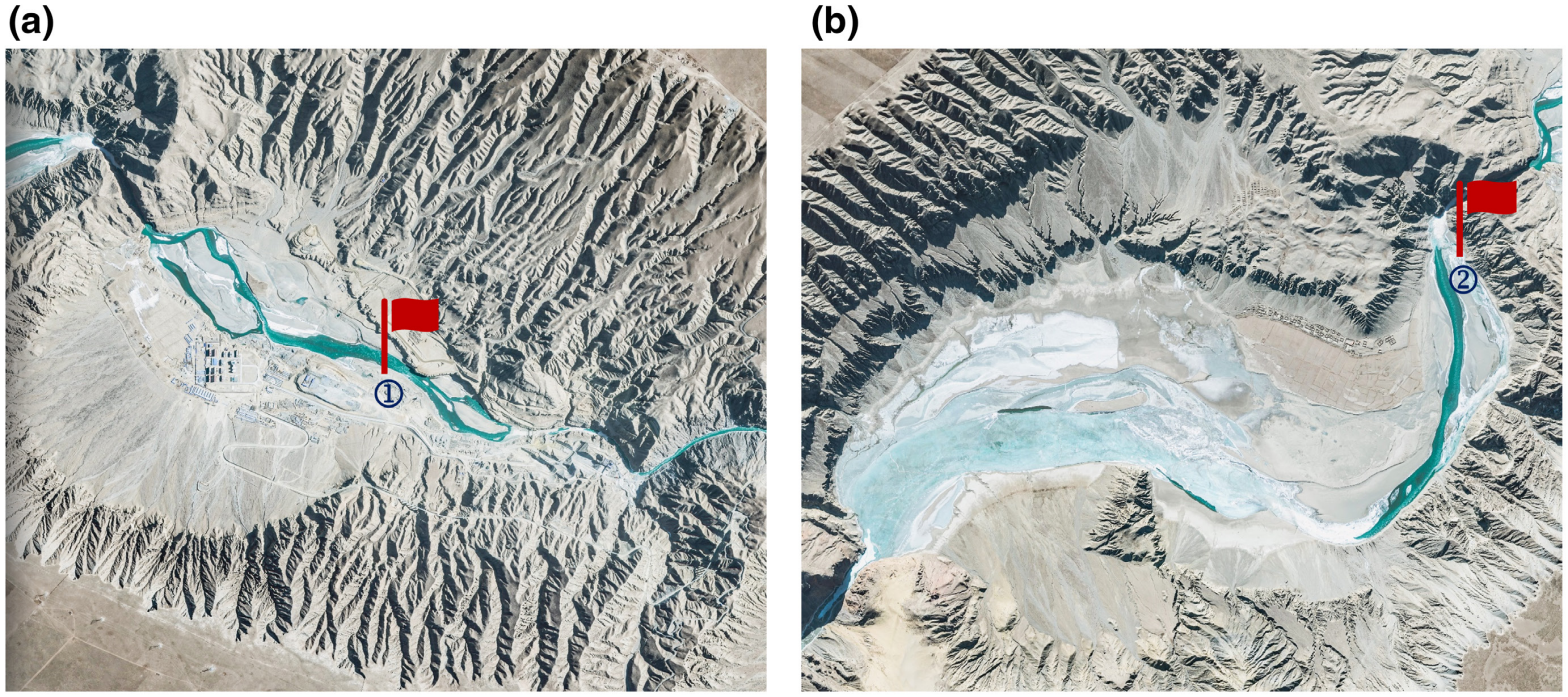
Spawning location (a) downstream of Yangqu Dam, (b) downstream of Yehu Gorge.

According to the results of our team's extensive survey in 2023, the spawning period of *G. eckloni* begins on May 19th and concludes on June 13th. The primary spawning locations are situated on the left bank below the Yangqu Dam and downstream on the left bank of the YeHu Gorge, denoted as points 1 and 2 in Figure 6. Specifically, point 1 is located at (35°43′49.63″ N, 100°16′27.47″ E), and point 2 is located at (35°45′7.51″ N, 100°17′43.74″ E). This study simulates the flow field during the spawning period below the Yangqu Dam based on the mentioned timeframe, providing an analysis of flow velocity and water depth at the main spawning locations. Subsequently, the downstream section of the YeHu Gorge is used as a validation area, employing the Habitat Simulation method to validate the obtained environmental factors.

### 3.2 Hydrodynamic factors

#### 3.2.1 Hydrodynamic model validation

To ensure the accuracy of the simulation calculations, model validation is crucial before conducting the simulations. This study employs a nonstructured mesh, with localized grid refinement in the primary spawning areas of fish. The grid nodes are set at a distance of 5 m in the fish's main spawning regions, while the rest of the areas have grid nodes set at a distance of 10 m, resulting in a total of 79,481 grids. The inflow boundary condition is set as flow rate, and the outflow boundary condition is set as water level. The hydrodynamic numerical model forms the data foundation for the fish habitat model. The model's roughness coefficient is adjusted to precisely emulate flow conditions in simulated scenarios, and this adjustment is then compared and validated against measured water level sections.

**FIGURE 7:**
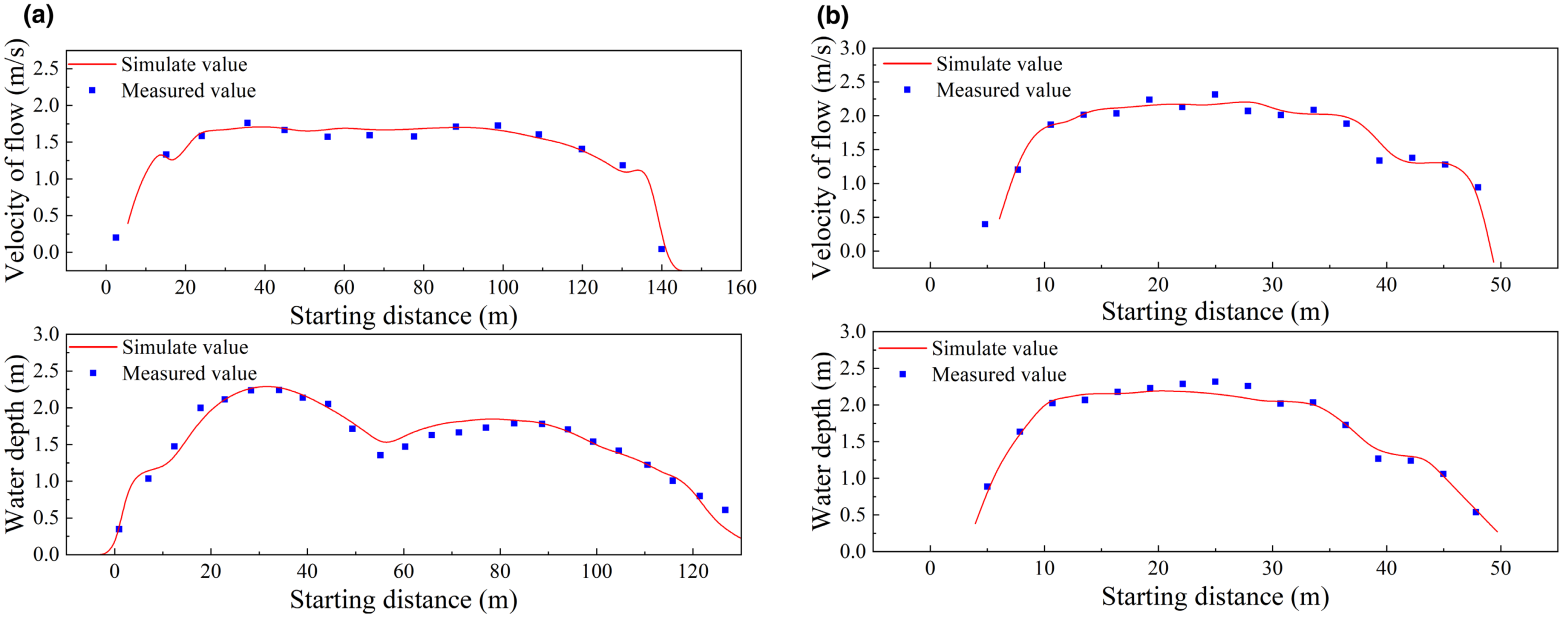
Comparison between measured and simulated values (a) 1# cross‐section, (b) 2# cross‐section.

The comparative analysis between the simulation results and measured values is presented in Figure 7, the model's calculated results align well with the measured data, with a maximum error of 5.8%. This indicates that the model has a good predictive capability for the flow conditions in the study area, demonstrating reliability and accuracy in the computed results.

#### 3.2.2 Hydrodynamic simulation results

The onset of spawning behavior occurs during the rising water process, and overall, the flow during the spawning period shows an increasing trend, consisting of three minor flood events. Within the 26‐day spawning period, 13 days experience rising water, 12 days see falling water, and 1 day remains stable. The maximum daily rise is 230 m^3^/s/d, the maximum fall is 124 m^3^/s/d, the average rise is 73 m^3^/s/d, and the average fall is 63 m^3^/s/d. Overall, the rise exceeds the fall in amplitude, with the minimum flow reaching 644 m^3^/s and the maximum reaching 1030 m^3^/s.

**FIGURE 8:**
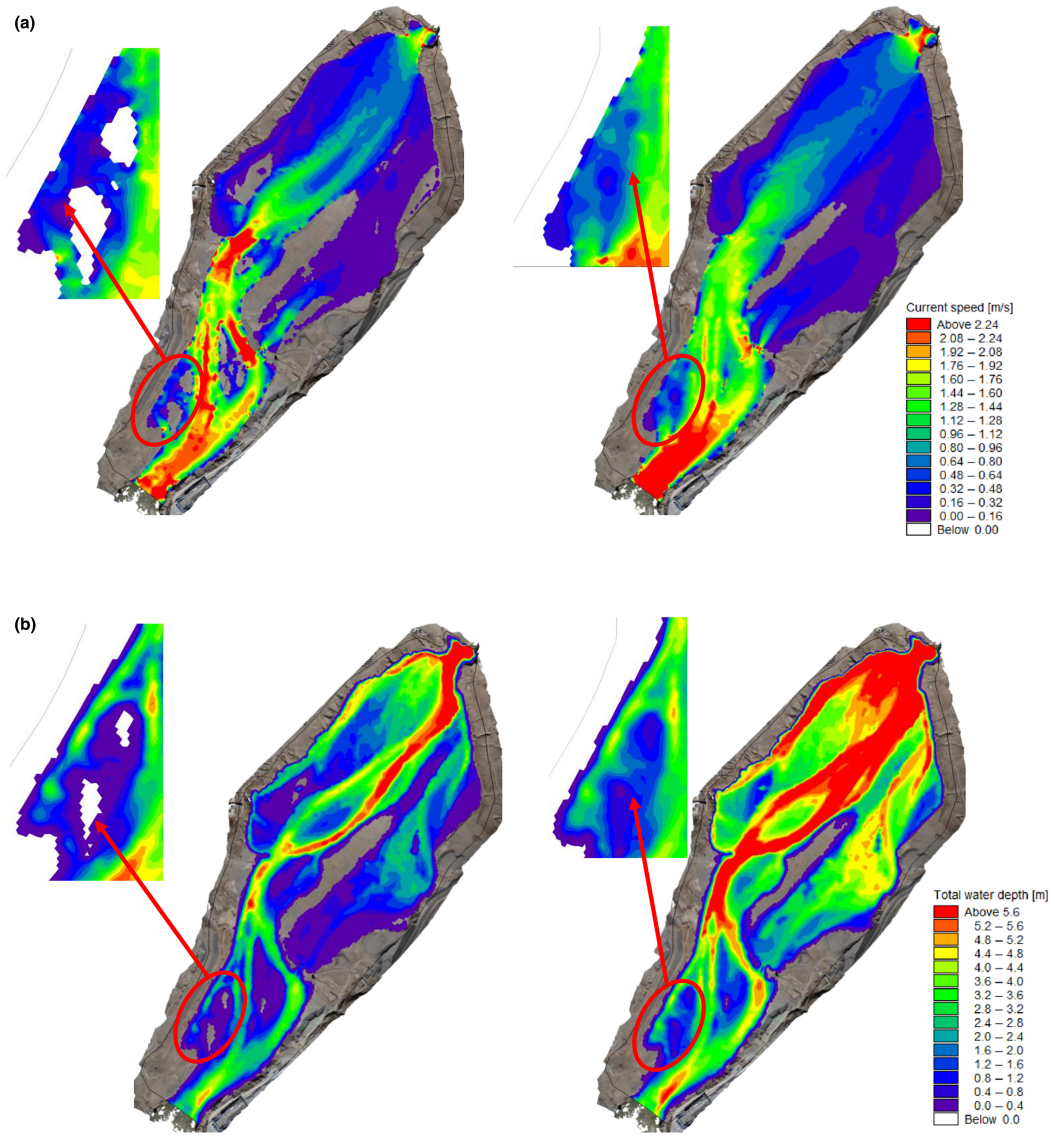
Numerical simulation results contour map (a) flow velocity, (b) water depth (Left: 644 m^3^/s, Right: 1030 m^3^/s).

To investigate the suitable flow velocity and water depth for *G. eckloni* spawning, numerical simulation is employed to calculate daily hydrodynamic conditions during the spawning period. Figure 8 illustrates a contour map of flow velocity and water depth for the minimum and maximum flow values at Yangqu Dam during the spawning period. It is evident that the mainstream in this area exhibits an S‐shaped morphology, accompanied by slow‐water zones on either side of the main flow. Past studies have often characterized these areas as slow‐water zones, defined by lower flow velocities. In the highlighted area on the left bank of the upstream, the flow velocity is lower, reaching around 0.2 m/s at a flow rate of 644 m^3^/s, and increasing to around 1 m/s at a flow rate of 1030 m^3^/s. Water depths vary from around 0.3 m to over 1 m. On the right bank downstream, there is a large area of low‐flow velocity, but in this region, water depths are mostly substantial, exceeding 2 m for most areas under low‐flow conditions and reaching above 3.2 m under high‐flow conditions. Greater water depths may pose challenges for fish spawning.

**FIGURE 9:**
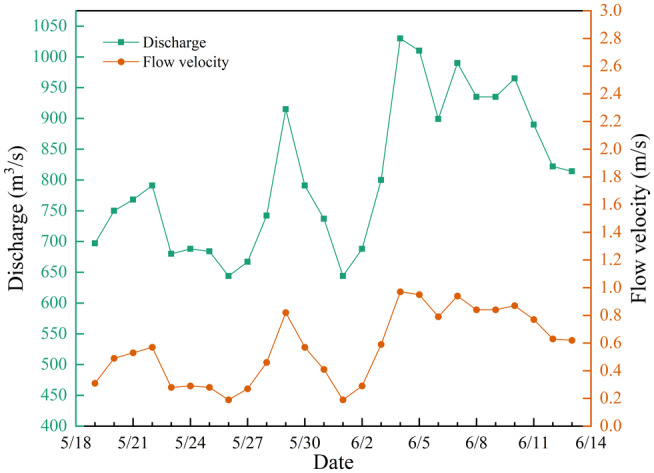
Spawning flow velocity line graph.

**FIGURE 10:**
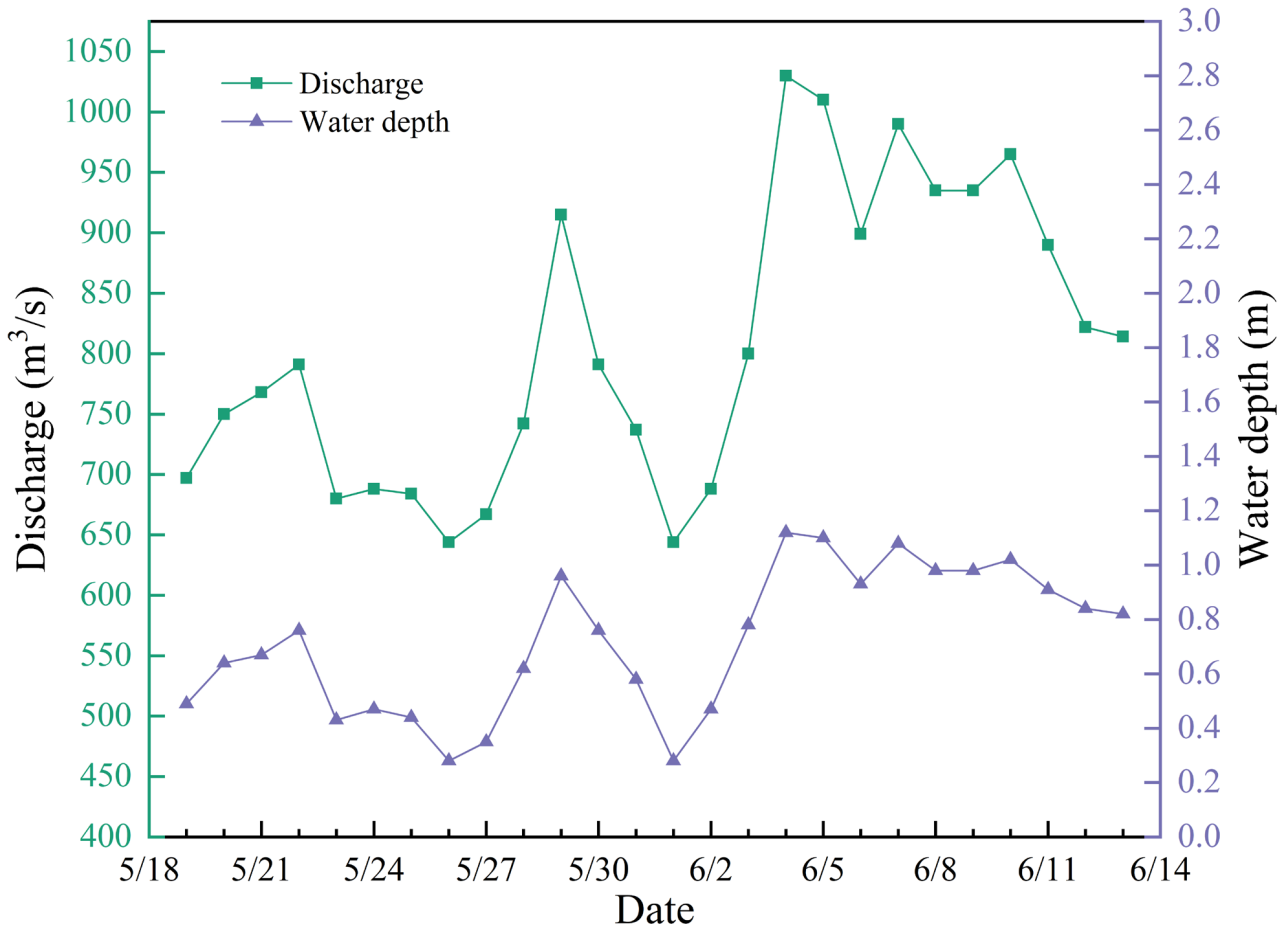
Spawning water depth line graph.

In the previous section, the primary spawning point in Yangqu Dam was identified as location 1, marked by the red circle in Figure 6. Subsequently, the flow velocities and water depths in this area were analyzed and averaged. Figures 9 and 10 illustrate the daily statistics of flow velocities and water depths. The chart indicates significant fluctuations in flow rates but relatively minor variations in flow velocities and water depths. The minimum recorded flow velocity is 0.19 m/s, and the maximum is 0.97 m/s. Similarly, the minimum water depth is 0.28 m, while the maximum is 1.12 m.

### 3.3 Water temperature

The monitoring device was completed and put into operation in March, with repeated calibrations performed on the water temperature probe before detection. A long sequence of hourly monitoring was conducted to assess the temperature requirements of *G. eckloni* during the spawning period. The average water temperature during the spawning period was 13.4°C, with a minimum daily average of 12.2°C and a maximum of 14.7°C. Throughout the spawning period, the water temperature exhibited an initial rise, a mid‐period decline, followed by a subsequent increase. The maximum daily temperature difference during the spawning period was 2°C, the minimum was 0.5°C, and the average was 1.1°C. The lowest water temperatures mostly occurred between 6:00 AM and 8:00 AM, while the highest temperatures were observed between 4:00 PM and 6:00 PM. These statistics highlight significant fluctuations in water temperature within the river segment. The traditional method of determining spawning water temperature using onsite measurements at a single time point is inadequate, as it fails to accurately encompass the true temperature requirements of the fish.

**FIGURE 11:**
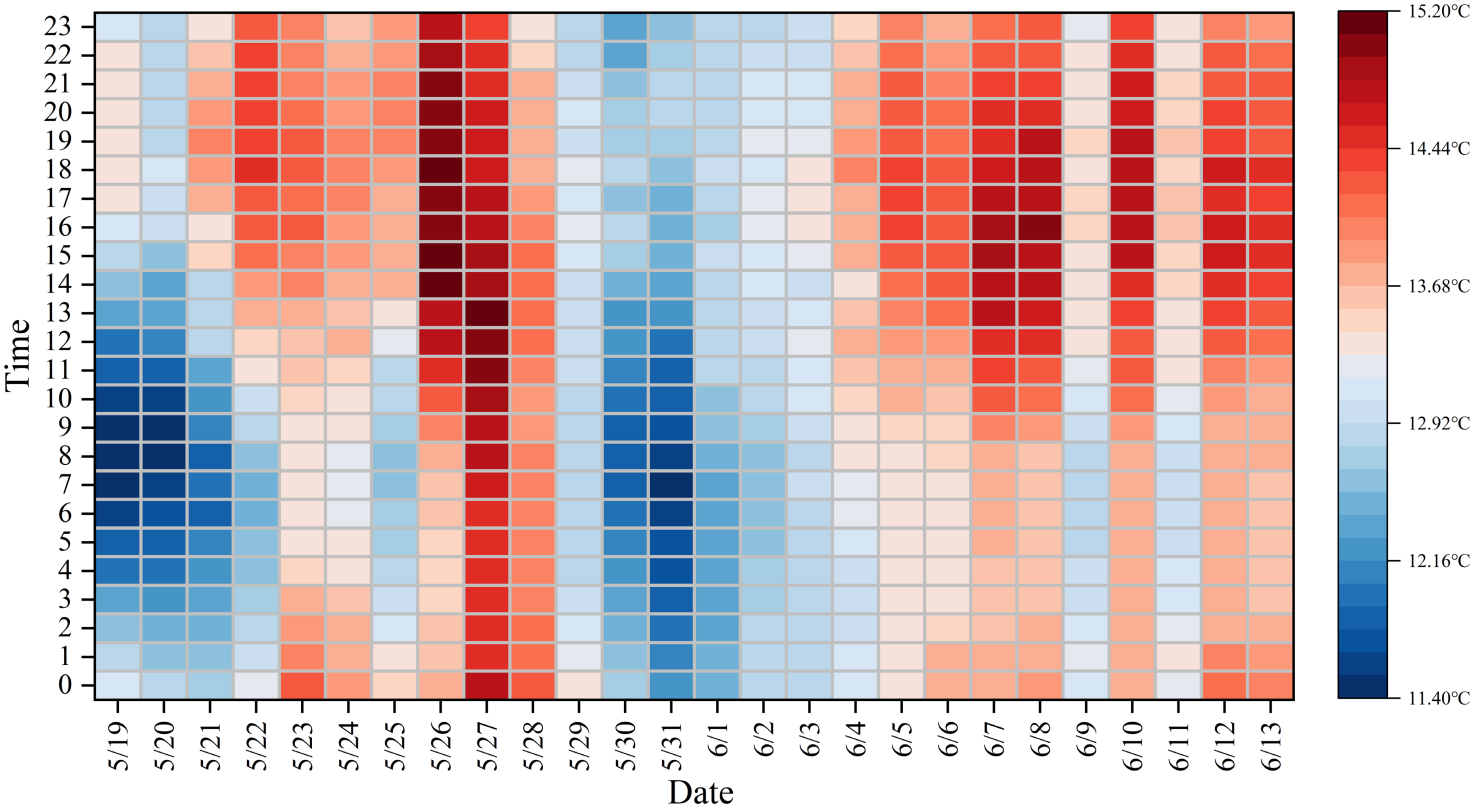
Hourly water temperature heat map.

Hourly data during the day (Figure 11) indicate that the minimum temperature during the reproductive period was 11.4°C, occurring at the beginning of the spawning period on May 19th at 8:00 AM, while the maximum temperature was 15.2°C, occurring in the mid‐spawning period on May 27th at 1:00 PM. These values span the entire temperature threshold range during the reproductive process, namely 11.4–15.2°C. This range provides a more scientifically accurate description of the spawning temperature requirements of *G. eckloni* compared to single‐timepoint and daily average measurements conducted onsite.

### 3.4 Substrate

**FIGURE 12:**
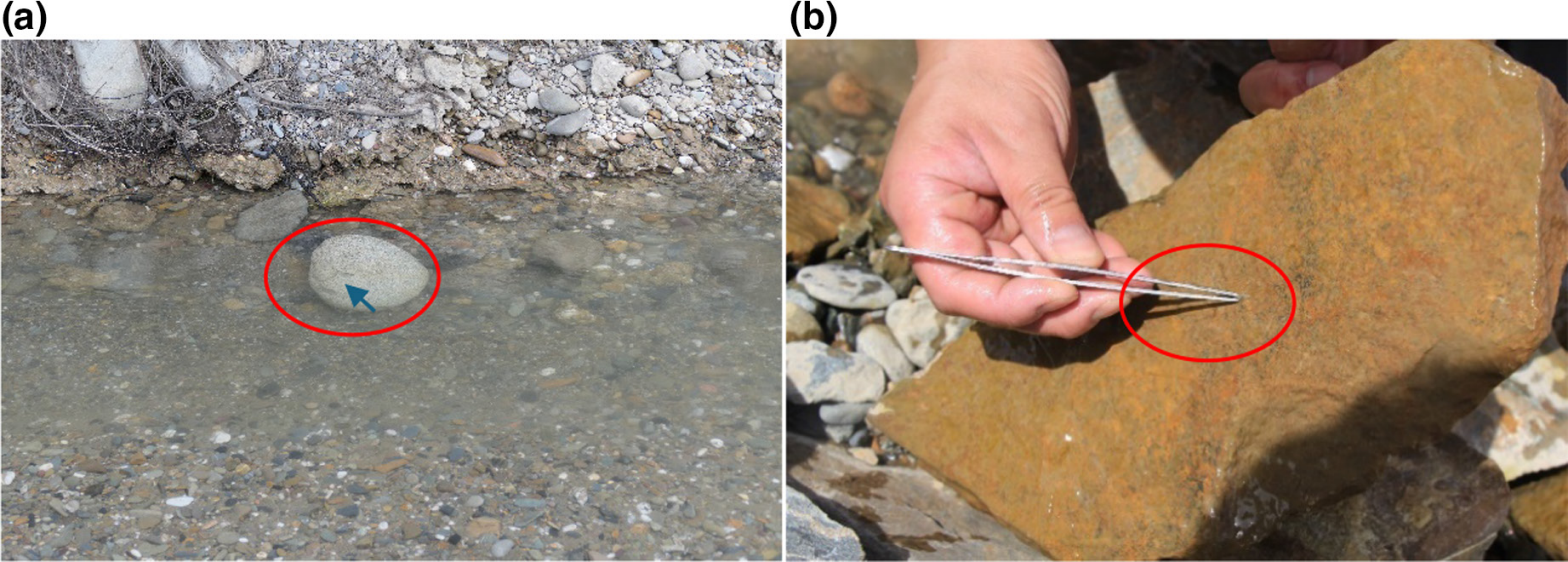
Spawning period substrate survey (a) 5/26, (b) 6/4.

Due to the poor water transparency of the Yellow River, it was often not possible to directly observe the spawning distribution in the spawning areas during the spawning period survey. However, some evidence was still obtained to demonstrate the spawning substrate of *G. eckloni*. Figure 12a shows fish eggs attached to round pebbles found in the still water area at the edge of the spawning region. Additionally, substrate excavation in the spawning area revealed fish eggs attached to the underside of a flat, square stone (Figure 12b). This indicates that the spawning substrate for *G. eckloni* consists of pebbles, which provide surfaces for egg adhesion, consistent with results obtained from laboratory experiments (Yan, 2016).

**FIGURE 13:**
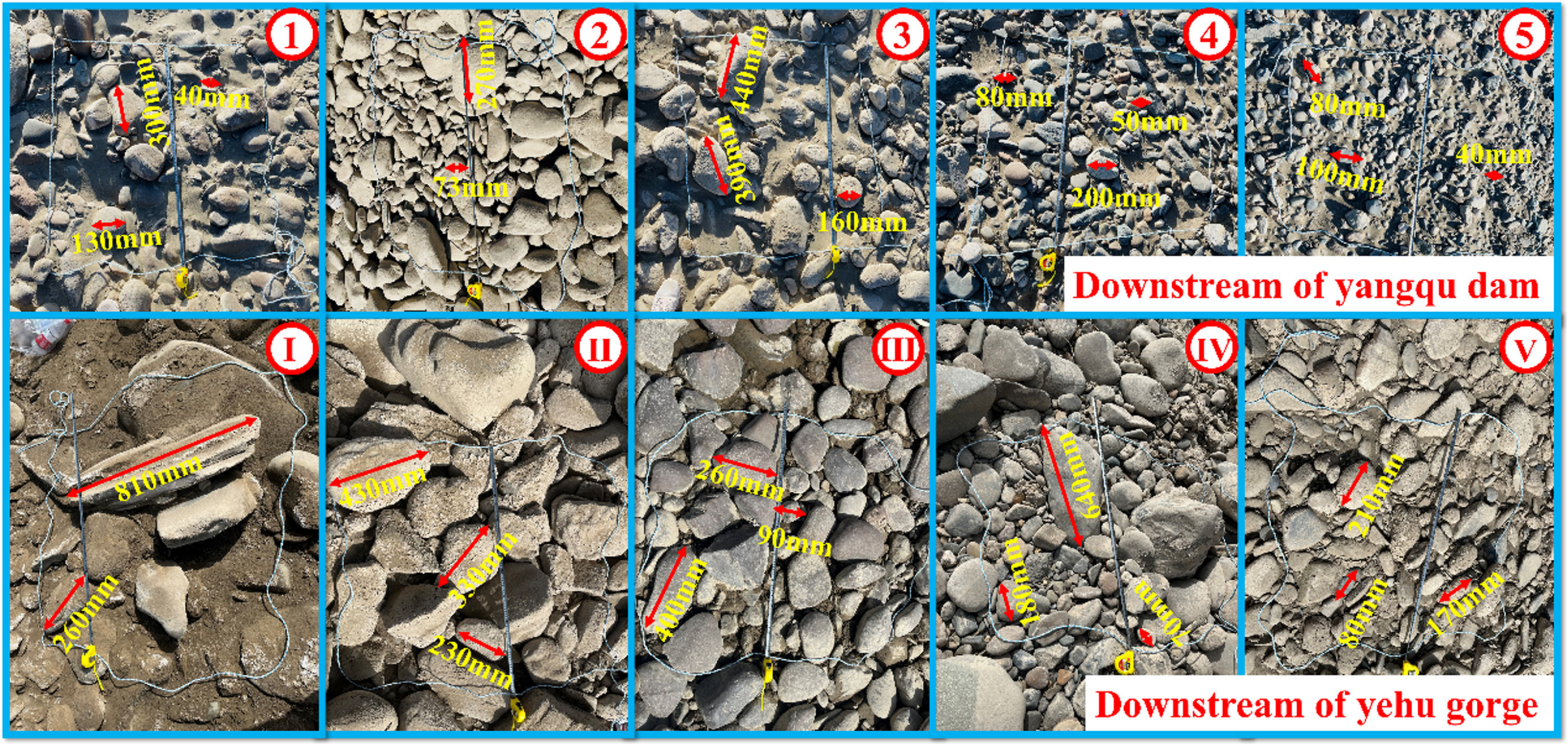
Substrate sampling analysis.

After confirming the spawning substrate for *G. eckloni* during the spawning period, the substrate conditions at the spawning locations were re‐evaluated during the low‐flow period. Although there were minor changes, the substrate remained primarily composed of pebbles suitable for egg adhesion. To obtain more detailed information about the substrate at the spawning grounds, multiple random sampling points were analyzed during the low‐flow period. Through exploration of the substrate during the low‐flow period at the spawning site (Figure 13), it was discovered that the primary composition of the substrate at the spawning site is predominantly gravel. To determine the composition ratio of the substrate, random samples were taken in the survey area using a 1 m × 1 m quadrat. The substrate types were classified based on the improved Wentworth substrate classification method proposed by Cummins (Cummins, 1962; Wentworth, 1922). Due to limitations in the sampling method and measurement accuracy, this study only classified gravel, categorizing it into Bowlder gravel (>256 mm), Cobble gravel (64–256 mm), and Pebble gravel (4–63 mm). Observations and measurements revealed that the maximum grain size of gravel downstream of the Yangqu Dam was 440 mm, while downstream of the Yehuxia Gorge it was 810 mm. The proportions of each type of gravel were then calculated, showing in the upstream region, giant gravel accounted for 20.1%, cobble gravel for 48.8%, and pebble gravel for 31.1%. In the downstream region, giant gravel accounted for 44.4%, cobble gravel for 46.7%, and pebble gravel for 8.9%. Combining the upstream and downstream data, the overall proportions were giant gravel 32.2%, cobble gravel 47.8%, and pebble gravel 20%. This data provides direct support for the subsequent restoration of the spawning ground.

### 3.5 Results validation

Habitat suitability modeling is commonly used in fisheries forecasting to predict fish catch at different locations based on various factors' suitability indices. In order to validate whether the results obtained from this method are reasonable, this study applies the obtained results to the downstream spawning ground of the YeHu Gorge. It is crucial to emphasize that since the survey period has reached the temperature threshold for spawning of *G. eckloni*, the water temperature suitability is assumed to be 1. The predominant substrate in the downstream area of YeHu Gorge is composed of gravel and sediment. Based on onsite survey results, the study classifies the substrate types in the area, with gravel areas being deemed suitable and other substrates as unsuitable.

**FIGURE 14:**
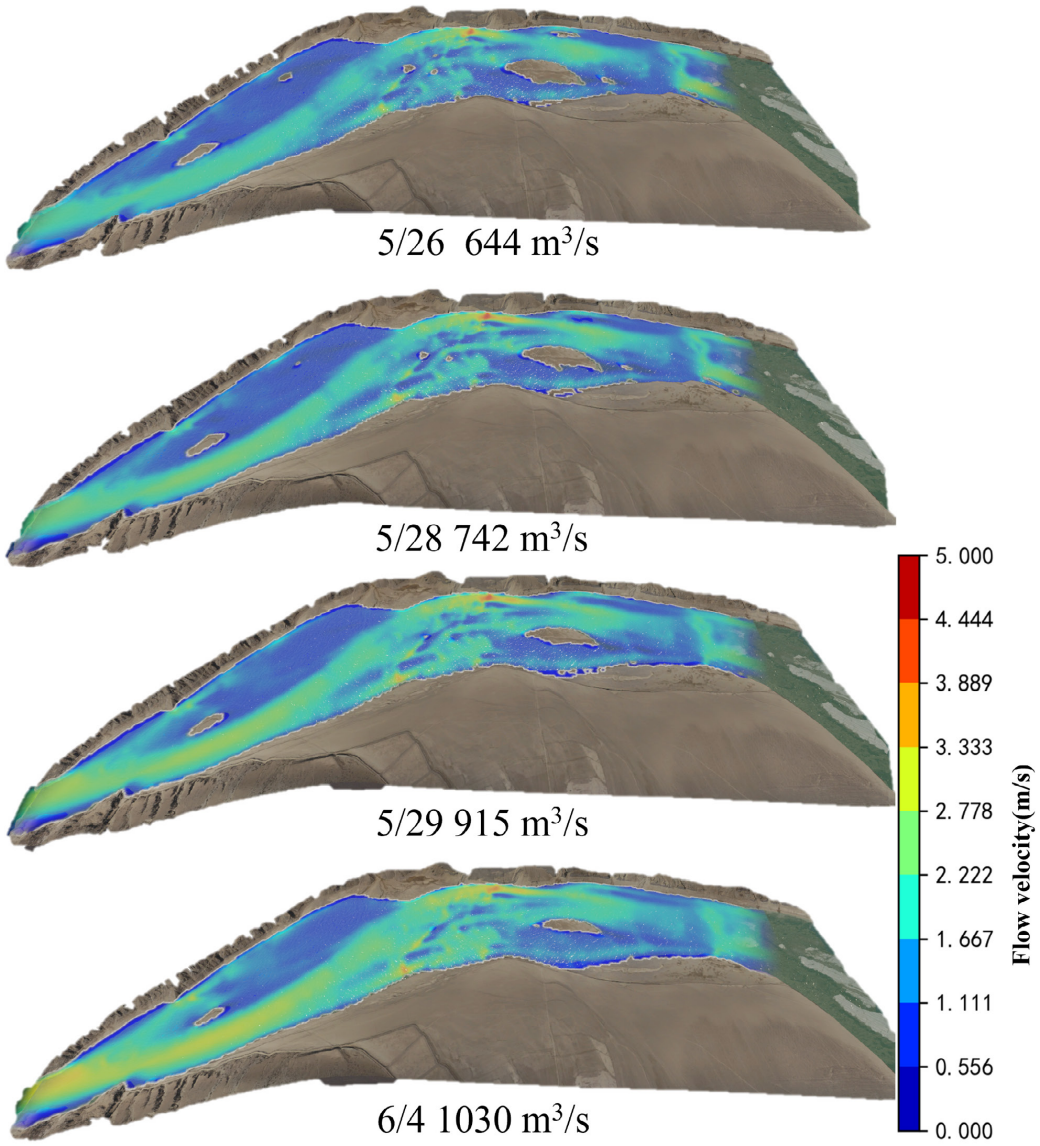
Flow velocity contour map.

**FIGURE 15:**
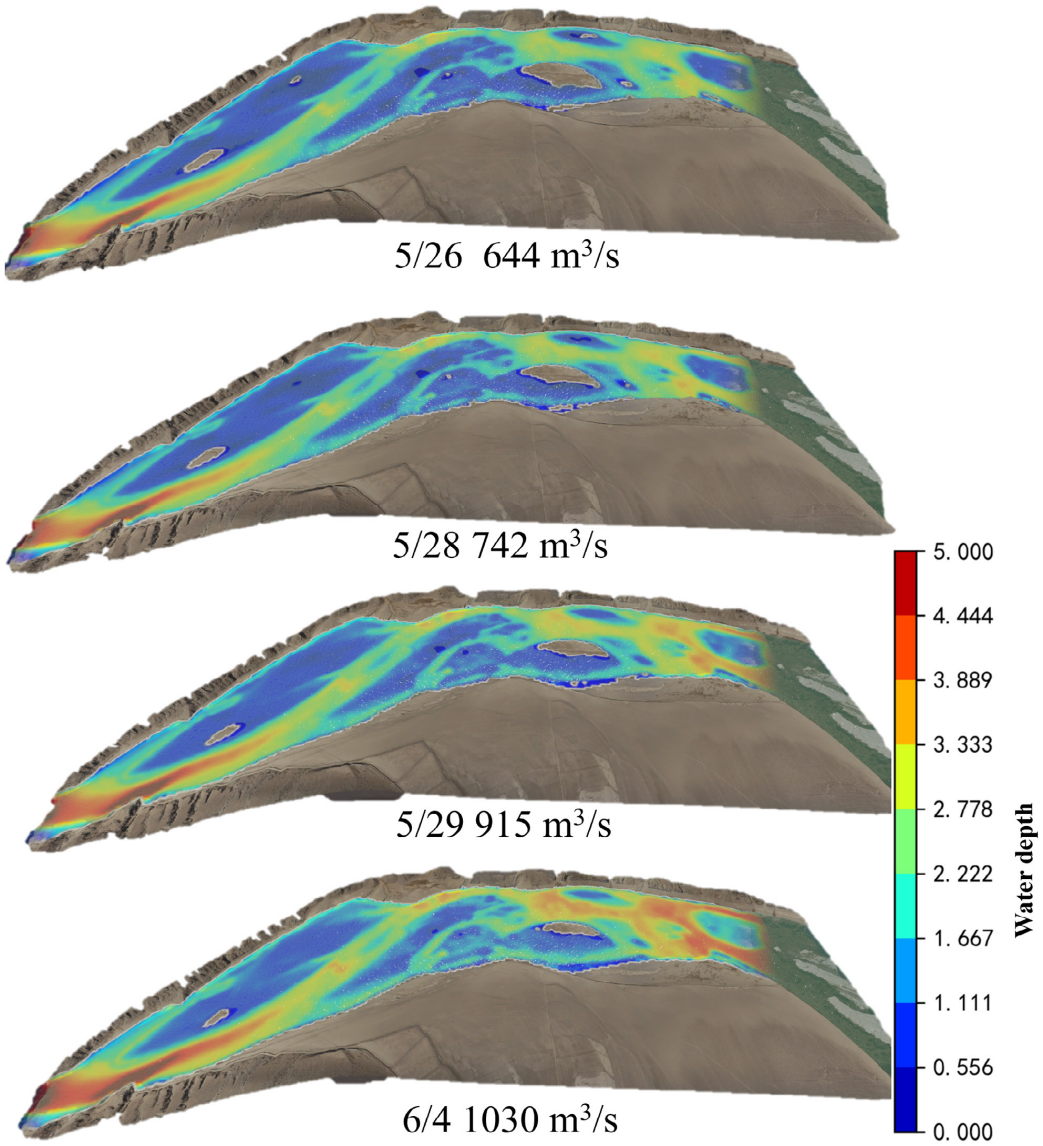
Water depth contour map.

**FIGURE 16:**
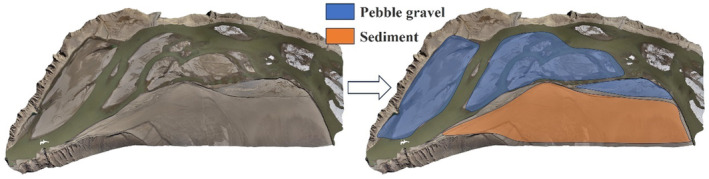
Substrate zoning.

The downstream validation conditions in YeHu Gorge are set similarly to those downstream of Yangqu Dam. Figures 14 and 15 display only partial calculation results. As depicted in the figures, water flows out from the exit of the YeHu Gorge. Due to the narrow and deep gorge, the main current at the exit exhibits high flow velocity and significant water depth. As the water progresses a certain distance, the main current tends toward the right bank, creating a slow‐flowing area on the left bank, represented by the blue area in the figures. Simultaneously, the substrate (Figure 16) in this area consists of gravel. In the downstream region, there is also a small blue slow‐flowing area, characterized by slow flow velocity and shallow water depth, with the substrate being suitable. Hence, based on the simulated results of flow velocity and water depth, combined with substrate conditions, the study calculates the suitability distribution of fish spawning in the area.

**FIGURE 17:**
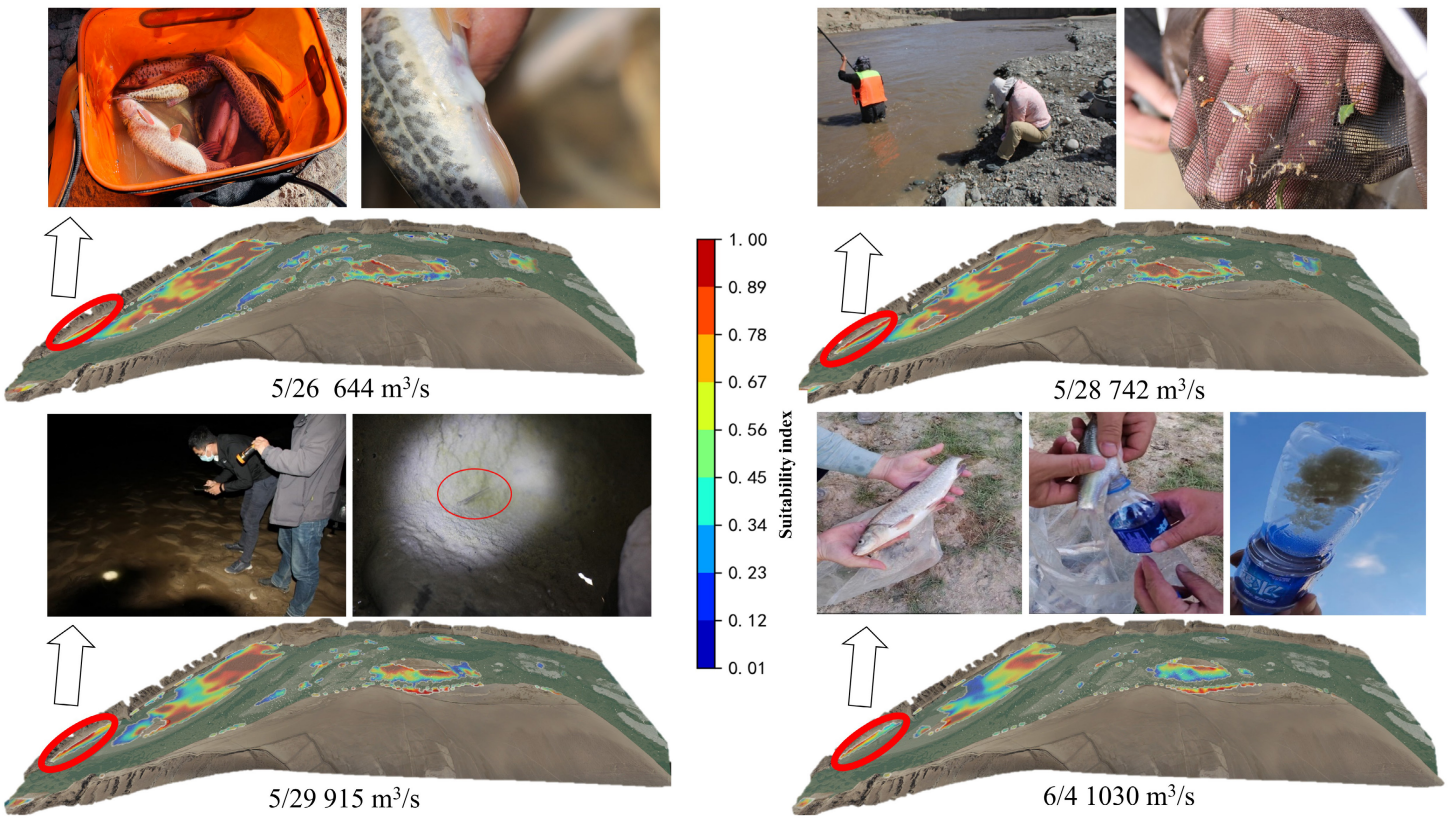
Suitability distribution contour map and field survey results.

Figure 17 presents the results of the simulation and the field investigation findings, showing that the suitable spawning areas for *G. eckloni* in this river segment are mainly divided into two parts: upstream and downstream. The upstream distribution is more concentrated, with a large and continuous high‐suitability area. In contrast, the downstream distribution is relatively scattered, with high‐suitability areas mainly concentrated near the right bank channel in the vicinity of the central island. It is noteworthy that the size and location of these suitability areas change with variations in flow and date, indicating the instability of the spawning ground. In comparison, the upstream area exhibits stronger stability. Additionally, we conducted long‐term monitoring of this region, focusing on whether mature fish entered the spawning period and whether *G. eckloni* fry were present in the area to verify that it serves as a spawning ground for *G. eckloni*. The survey results show that on May 26, 11 *G. eckloni* were caught using a fishing net, and upon light pressure on their abdomens, one male fish released sperm and two female fish released eggs. On May 28, multiple *G. eckloni* fry were found in the area using a dip net. On the night of May 29, an investigation revealed swimming fry in the still water areas at the edge of the region. Additionally, on June 4, eight *G. eckloni* were caught using a fishing net, and upon light pressure on their abdomens, eggs were released from three female fish. The above investigations in this region discovered mature adult fish with developed gonads and about to spawn, and at the same time collected newly born fry. This is sufficient to prove that this region is a spawning ground for *G. eckloni*, which is the No. 2 spawning site confirmed by this field investigation. Combined with our simulation results, which show strong suitability at this location, this confirms that the identified environmental factors necessary for *G. eckloni* spawning are accurate. Of course, other high‐suitability areas may still be potential spawning sites; however, due to environmental and other limiting factors, investigations of these areas were not conducted. These locations will be key focus areas for future research.

## 4 CONCLUSION AND DISCUSSION

In the study of natural reproductive ecological requirements, environmental parameters such as water temperature, water flow, water depth, and riverbed quality are universally considered factors (Seesholtz et al., 2015). The impact of environmental factors on fish reproduction is a comprehensive and complex outcome, and the differences and variations in environmental factors can either promote or inhibit natural fish reproduction. Therefore, different fish species require specific environmental factors to engage in spawning activities. Generally, the reproductive activities of fish are not determined by a single environmental factor; their specific reproductive requirements often result from the combined influences of multiple environmental factors. This study employs a systematic and scientific approach to report, for the first time, the spawning requirements of the protected fish species, *G. eckloni*, in the upper reaches of the Yellow River. By identifying the spawning period and locations through surveys and employing various methods, the key ecological factors required for the spawning of *G. eckloni* were clarified. These factors include a range of flow velocities (0.19–0.97 m/s), water depths (0.28–1.12 m), water temperatures (11.4–15.2°C), and substrate types primarily composed of gravel. The validity of these indicators was further confirmed by applying them to the downstream section of YeHu Gorge.

Previous studies on the reproductive ecological requirements of *G. eckloni* have been limited, with most focusing on artificial breeding and resource distribution. Sili Yan provided a qualitative description of its reproductive‐related aspects, suggesting that the reproductive season for *G. eckloni* generally occurs from April to May, sometimes extending to June or July. The peak spawning period was noted to be in late May (Yan, 2016). The present study's investigation confirmed the spawning period for *G. eckloni* to be from late May to early June, lasting less than a month, aligning with the previously described timeframe.

Water temperature has a dual impact on the spawning time of fish. First, sufficient accumulated temperature promotes the maturation of fish gonads, and second, after gonadal maturation, there is a threshold water temperature for spawning (Li et al., 2021). The study identified the spawning threshold water temperature for *G. eckloni* as 11.4–15.2°C. This aligns closely with the recommended temperature range of 14–16°C for artificial breeding (Dong et al., 2016). The slight difference in the starting temperature is primarily due to the need for artificial breeding to set the water temperature optimally, while in the upper reaches of the Yellow River, the water temperature during the spawning period rises gradually from low to high. Experimental studies have shown that the hatching rate of *G. eckloni* fertilized eggs increases with rising water temperature, peaking at 25°C and then declining (Yan, 2016). During the study period, the water temperature was in a rising phase, consistent with the research findings. After the spawning period of *G. eckloni*, as the water temperature continues to rise, it promotes the hatching and embryonic development of fertilized eggs, providing additional evidence for the validity of the water temperature range obtained in this study.

Flow velocity and water depth play a crucial role in ensuring the exchange of substances, oxygenation, and protection against UV radiation for fish egg incubation (Hui et al., 2012; Zhou, 2023). These factors are essential for habitat assessment and conservation. Many scholars have used expert experience methods (Cai et al., 2012; Han et al., 2011; Wang et al., 2023), indoor flume experiments (Gong et al., 2015; Shi et al., 2012; Yang et al., 2007), and field measurement methods (Gao et al., 2020; Yang, 2007; Zhang et al., 2007) to obtain the necessary data. Due to the difficulty in acquiring ecological data, the expert experience method has been applied to various fish species in multiple river basins (Zhao et al., 2013). Some scholars have estimated the spawning water depth requirement for *G. eckloni* to be around 1 meter (Yan, 2016) and the flow velocity to be 0.3–0.8 m/s (Li et al., 2007), which is similar to the results of this study. However, these results are inevitably influenced by subjective expert experience. The field measurement method is the most accurate way to reflect the true needs of fish, but due to equipment limitations, hydrodynamic characteristics within the area can only be measured at single points (Peng et al., 2018). Indoor experimental methods have become the mainstream approach in recent years for understanding fish needs (Chen et al., 2021; Li et al., 2015), as they can depict the response mechanisms of fish to different flow velocities and depths. However, due to differences between indoor and outdoor environments, fish may experience stress reactions, leading to results that differ from their behavior in natural conditions. This study combines field measurements and numerical simulations, matching factors, and methods to obtain more scientific and accurate results. By using precise spawning patch locations instead of the spawning sections used in previous studies and applying numerical simulation methods to account for the overall characteristics of the spawning area, the study determined that the spawning requirements are a flow velocity of 0.19–0.97 m/s and a water depth of 0.28–1.12 m. This represents a significant advancement in scale and overcomes the shortcomings of previous methods, demonstrating strong scientific accuracy.

Most indigenous fish species in the upper reaches of the Yellow River lay adhesive eggs, and cobble gravel substrates are the primary sites for adhesion and incubation (Zhao et al., 2020). This study corroborates these findings and provides a statistical analysis of the proportion of cobble gravel substrates, offering valuable data support for future restoration efforts in spawning areas.

Only with sufficient understanding can effective conservation be achieved. This study, based on the investigation conducted in the current year, employed habitat simulation methods to validate the results and also drew comparisons with historical studies on the reproductive ecological requirements of *G. eckloni*. This significantly enhances the credibility of the findings. However, continuous research progress is still required through ongoing long‐term monitoring and investigations. Additionally, factors influencing fish spawning extend beyond the parameters proposed in this paper, such as environmental noise, water quality conditions, and light exposure, which may also have significant impacts on the spawning behavior of *G. eckloni*. The roles and interrelationships of these factors need further exploration and clarification in subsequent long‐term studies.

## AUTHOR CONTRIBUTIONS

**Lihao Guo:** Conceptualization (equal); data curation (equal); methodology (equal); writing – original draft (equal). **Guodong Li:** Investigation (equal); methodology (equal); visualization (equal). **Qiaoling Zhang:** Resources (equal); supervision (equal). **Guoyong Zhang:** Supervision (equal). **Weiying Wang:** Project administration (equal). **Zijun Liu:** Visualization (equal). **Shanshan Li:** Formal analysis (equal).

## FUNDING INFORMATION

The authors declare that no funds, grants, or other support were received during the preparation of this manuscript.

## CONFLICT OF INTEREST STATEMENT

The authors have no relevant financial or nonfinancial interests to disclose.

## Data Availability

The data for this paper have been uploaded to https://datadryad.org/stash/share/fbtBDEgsAnOib_TftXGfmdX23QZrZJvfXCDbMWbpJWg.
